# Understanding Final Neolithic communities in south-eastern Poland: New insights on diet and mobility from isotopic data

**DOI:** 10.1371/journal.pone.0207748

**Published:** 2018-12-19

**Authors:** Anita Szczepanek, Zdzislaw Belka, Paweł Jarosz, Łukasz Pospieszny, Jolanta Dopieralska, Karin M. Frei, Anna Rauba-Bukowska, Karolina Werens, Jacek Górski, Monika Hozer, Mirosław Mazurek, Piotr Włodarczak

**Affiliations:** 1 Institute of Archaeology and Ethnology, Polish Academy of Sciences, Cracow, Poland; 2 Isotope Laboratory, Adam Mickiewicz University, Poznań, Poland; 3 Institute of Archaeology, Adam Mickiewicz University, Poznań, Poland; 4 Institute of Archaeology and Ethnology, Polish Academy of Sciences, Poznań, Poland; 5 Poznań Science and Technology Park, Poznań, Poland; 6 National Museum of Denmark, Copenhagen, Denmark; 7 Department of Anatomy, Jagiellonian University Medical College, Cracow, Poland; 8 Department of History and Cultural Heritage, University of Pope Jan Paweł II, Cracow, Poland; 9 Institute of Archaeology, University of Rzeszów, Rzeszów, Poland; Museo delle Civiltà, ITALY

## Abstract

We present the first comprehensive multi-isotopic data on human and animal remains from the Final Neolithic Corded Ware culture (ca. 2900–2300 cal. BC) in south-eastern Poland. The study focused on communities of two settlement areas located in the Małopolska Upland and in the Subcarpathian region. Carbon and nitrogen isotopes of bone collagen were investigated to obtain insights into human dietary preferences, whereas the strontium isotope composition of human tooth enamel was used to trace the mobility and provenance of individuals. Sr isotope data point to a non-local origin of at least one-quarter of the investigated individuals in the Subcarpathian region, consistent with associated allochthonous grave inventories of eastern or western origins. In contrast, all investigated individuals in the Małopolska Upland were of local origin. Furthermore, our study shows an example that the use of fauna for the assessment of the local ^87^Sr/^86^Sr range of an archaeological site can lead to incorrect conclusions and suggests that a detailed Sr isotopic survey of the geological background and its hydrologic elements is necessary to provide conclusive constraints for the identification of local and non-local individuals in prehistoric communities. Carbon and nitrogen isotope composition of bone collagen indicate an omnivorous diet that included C_3_-based terrestrial plant and animal resources, in which plant food dominated. In both regions, there were no significant sex differences in dietary intakes. Higher δ^15^N_coll_ values of younger infants presumably reflect the effect of weaning.

## Introduction

Measurements of stable and radiogenic isotopes in human and animal tissues are a useful tool in archaeological research to enrich knowledge about past populations [1, 2, 3, 4, 5]. Carbon and nitrogen isotope compositions of bone collagen provide proxies that allow reconstruction of dietary patterns and, in some cases, proportions of terrestrial vs. aquatic resources [6] and the use of C_3_- vs. C_4_-based foods within terrestrial ecosystems [7]. In addition, δ^15^N_coll_ values correspond to trophic levels that organisms have in the food chain (e.g. [8]). Strontium isotope ratios (^87^Sr/^86^Sr) of tooth enamel enable identification of “local” vs. “non-local” members of a community and thus allow tracking of the mobility of individuals from the studied sites [9, 10, 11, 12] because strontium is fixed during enamel mineralization and the ^87^Sr/^86^Sr values reflect the Sr isotope composition of the environment where an individual lived when the enamel mineralization occurred. A local range of ^87^Sr/^86^Sr values is often defined as ±2 s.d. from the mean ^87^Sr/^86^Sr in tooth enamel from prehistoric animals that lived locally (e.g. [13, 14, 15]). Recently, however, Burton and Hahn [16] expressed the opinion that this method can lead to “wrong, even absurd, inferences of locality and mobility”. In addition, another recent study by Grimstead et al. [17] demonstrates that a standardization for the collection of necessary baseline environmental data would be necessary, which does not currently exist. Therefore, the present study aimed also to provide some insights into this issue by investigating different strontium sources in the area studied, namely faunal remains, local bedrock and atmospheric waters.

**Fig 1:**
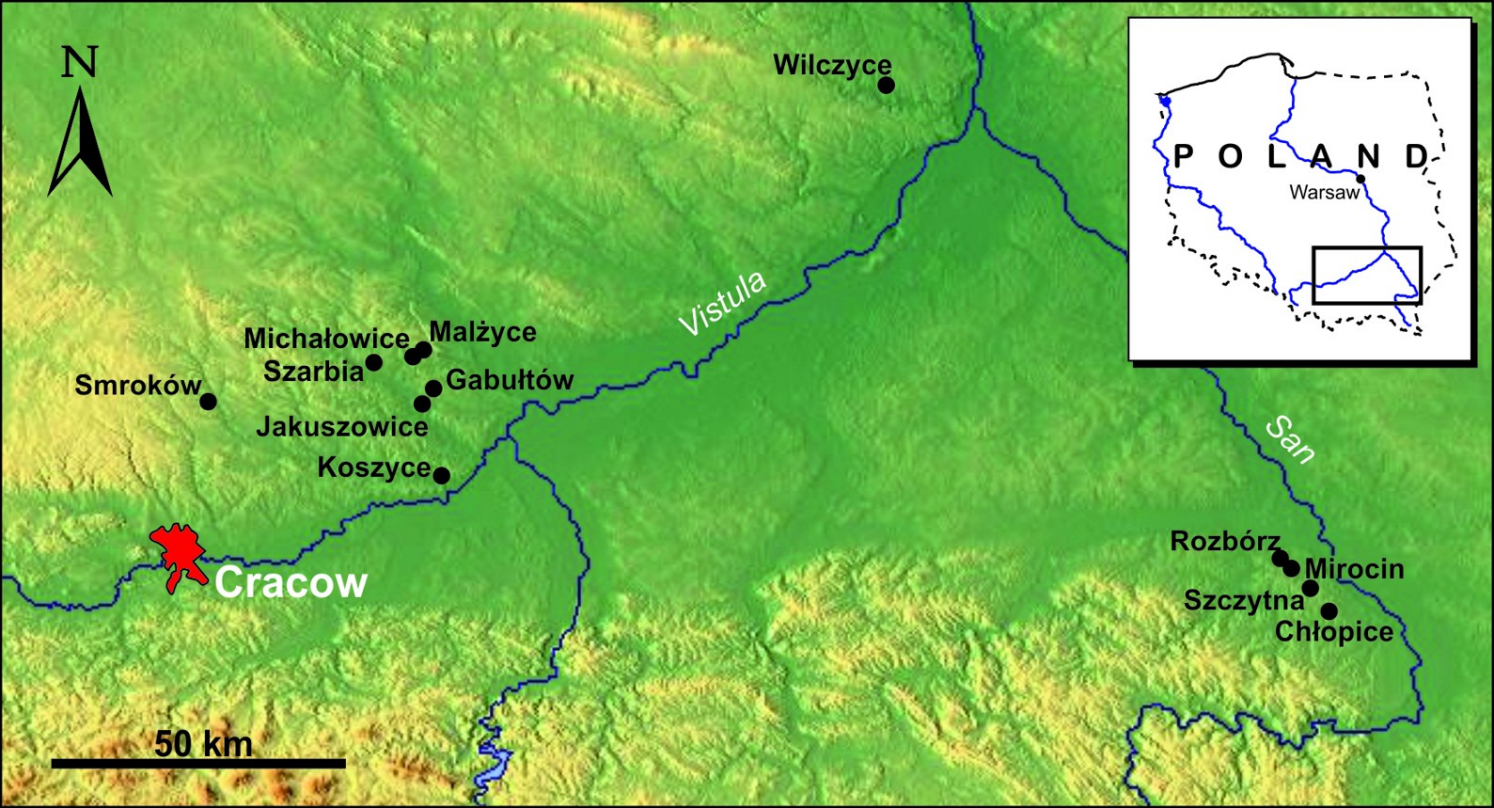
Map of south-eastern Poland with the location of archaeological sites in which the human and animal remains were investigated for the present study. Inset shows the location of the study area in Poland.

The aim of this paper is to present the results of a multi-isotopic study of the Final Neolithic Corded Ware culture (CWC) populations from the uplands of south-eastern Poland. Investigations have been concentrated in two settlement regions—one in the Małopolska Upland and the other in the Subcarpathian region (Fig 1).

In this part of Central Europe, the Final Neolithic was a time of cultural and social changes connected with the origin and development of CWC communities [18, 19]. Previous isotopic studies in the area focused on oxygen isotope data from single archaeological sites only [20, 21]; hence, this work provides the first comprehensive multi-isotopic survey for CWC communities in south-eastern Poland.

**Fig 2:**
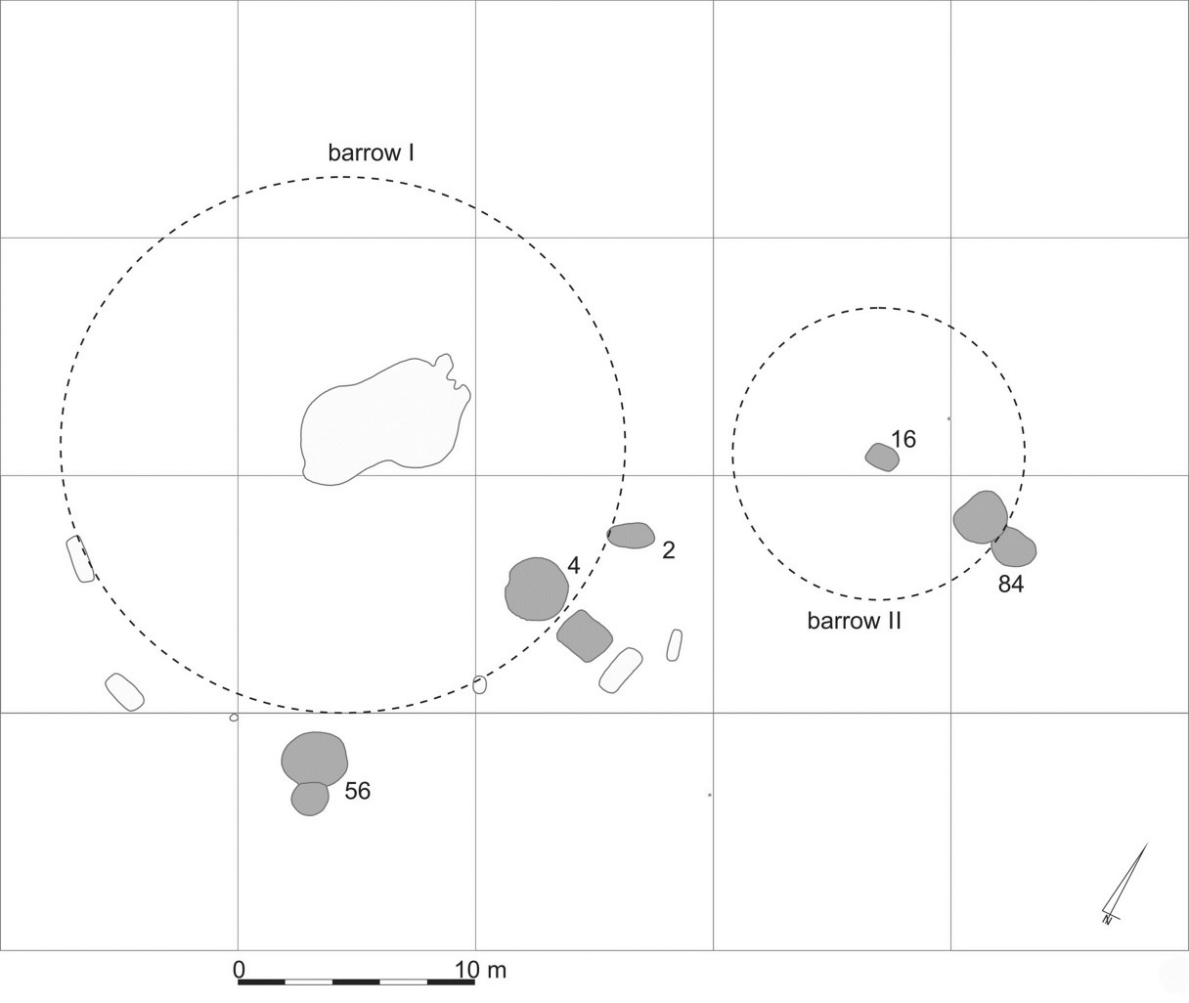
The configuration of graves at the analysed site 6 in Szczytna.

The archaeological record revealed that CWC societies in the Małopolska Upland and in the Subcarpathian region followed similar funeral rites. In the older phase of CWC development (28th-26th century BC), the deceased were buried mostly in central pits under mounds [22]. In the central grave under the mound, the body arrangement of the inhumations and grave furnishings were consistent with the model emphasizing the sex and age of the buried individuals [23]. In the younger phase of CWC development (25th-23rd century BC), these mounds became sacred places around which graves with a niche construction were created (Fig 2).

**Fig 3:**
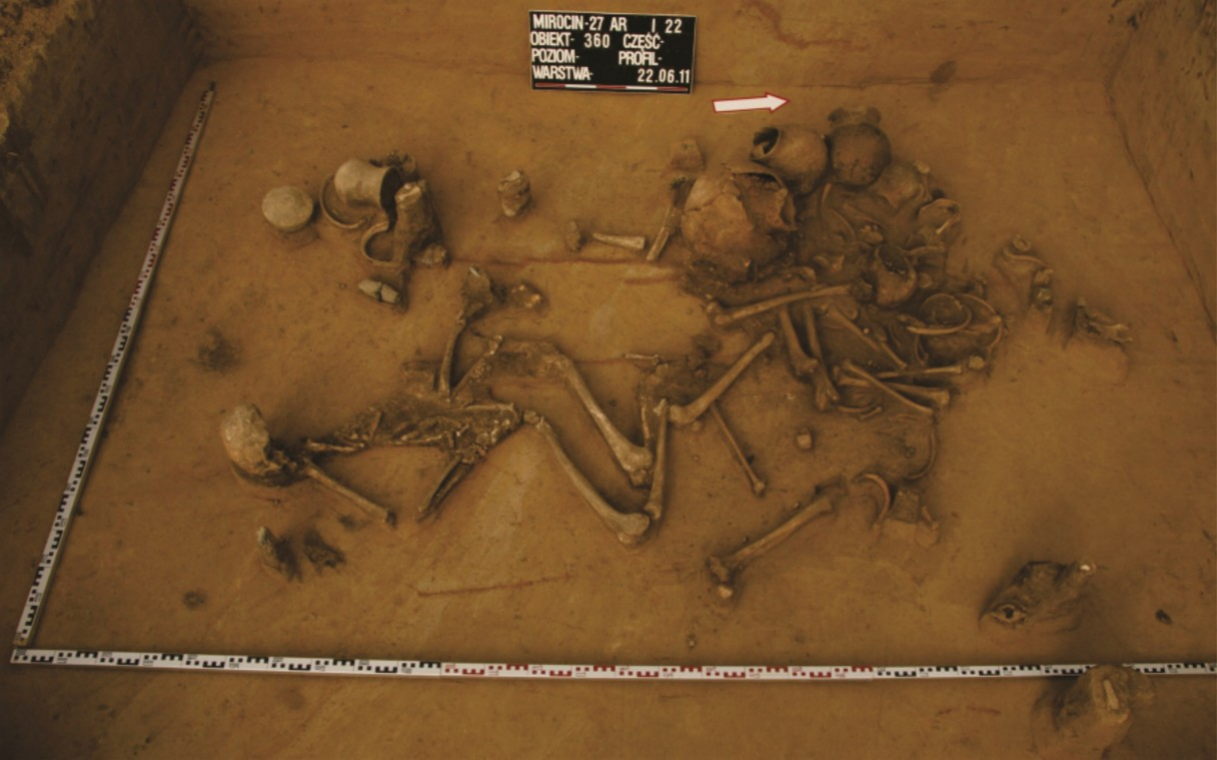
Double grave no. 360 from Mirocin, site 27 –distribution of skeletons and grave goods.

The features included an entrance pit, passage and grave chamber. As a rule, the burials were richly furnished with pottery vessels, bone and lithic inventories, and sometimes copper objects (Fig 3).

The male skeletons rested on their right side, accompanied by distinctive offerings, which were objects regarded as markers of gender and social status, such as stone battle-axes, flint axes and flint arrowheads. Apart from this type of burial, there were also graves with the body resting on its left side and furnished with pottery vessels and singular tools only. Most individuals resting on their left side were females, with only a small number of males found in this position [24]. This special funeral rite with barrows marking the boundary of the territory penetrated by a given human group and no stable settlements depict the CWC as an archaeological relict of a pastoral socio-economic formation [25]. A typical feature of such societies is a periodic mobility and sometimes migration in search of new pastures [26, 27, 28, 29]. Consequently, members of the CWC communities are targets of interest in isotopic research.

## Materials

Graves from the Małopolska Upland and Subcarpathian region have a similar construction type, and most of these graves surround mounds dated to the Middle or Final Neolithic age (Fig 2). The graves of the Małopolska Upland were discovered within a zone intensively occupied by CWC communities. Sites attributed to this culture have been excavated since the beginning of the 19^th^ century. In contrast to Małopolska, the Final Neolithic occupation of the Subcarpathian region remained poorly recognized until the last decade. Recent discoveries were made during excavations preceding motorway construction [30]. The present study included skeletal remains collected from the following sites:

### Małopolska Upland

**Malżyce, site 30** (50^o^22’46”N; 20^o^29’54”E): remnants of an extensive tumulus cemetery initiated by the Funnel Beaker Culture (FBC) community and later reused in the CWC period. Tumulus 1 of FBC was encircled by CWC niche graves 2, 4, 5, 7 [31] and tumulus 2 was surrounded by niche graves 10, 11, 12 [32]. All these graves contained single burials of individuals of different ages and sexes [33]. The material is housed in the collections of the Institute of Archaeology and Ethnology, Polish Academy of Sciences.

**Gabułtów, site 1** (50^o^16’59”N; 20^o^31’10”E) is a barrow cemetery of CWC comprising an older central grave (feature 1) covered by a mound and a younger secondary triple burial in the niche grave (feature 2) dug into its surroundings [34, 35]. The mound was later reused by the Trzciniec culture community dated to the Older Bronze Age. The material is housed in the collections of the Archaeological Museum in Cracow.

### Subcarpathian region

**Szczytna, site 5** (50^o^0’28”N; 22^o^35’50”E) is a multicultural site occupied from the Neolithic to Early Bronze period and then in the Modern Period. The CWC graves were of a niche construction with single (no. 217) or double burials (no. 220) [36]. The material is housed in the collections of the Regional Museum in Rzeszów.

**Szczytna, site 6** (50^o^0’43”N; 22^o^35’39”E) is a multicultural site occupied from the Neolithic to the Early Bronze period and then in the Modern Period. The CWC single niche graves (4, 56, 84, 150) surrounded the earlier barrow dated to the older phase of the culture [36]. The material is housed in the collections of the Regional Museum in Rzeszów.

**Mirocin, site 24** (50^o^1’53”N; 22^o^34’28”E) is a multicultural site occupied from the Final Neolithic to Early Bronze period and then in the Modern Period. The CWC niche graves (50, 54, 110) surrounded the earlier barrow dated to the older phase of the culture [30]. The material is housed in the collections of the Rzeszów Archaeological Centre Foundation.

**Mirocin, site 27** (50^o^2’40”N; 22^o^34’17”E) is a multicultural site occupied from the Neolithic to Early Bronze period and then in the Modern Period. The CWC grave was a niche construction and contained remains of two individuals [37]. The material is housed in the collections of the Rzeszów Archaeological Centre Foundation.

**Rozbórz site 42** (50^o^4’18”N; 22^o^33’5”E) is a multicultural site occupied from the early Neolithic to Modern Period. The CWC single burial of a male was discovered in a pit grave [30]. The material is housed in the collections of the Rzeszów Archaeological Centre Foundation.

**Chłopice, site 26** (49^o^57’26”N; 22^o^39’37”E) is a multicultural site occupied from the Neolithic to Early Bronze period. The CWC grave was of a niche construction and contained the remains of two individuals [30]. The material is housed in the collections of the Rzeszów Archaeological Centre Foundation.

Individuals buried in these graves were subjected to multi-isotopic analyses. In most cases, analyses of δ^13^C, δ^15^N and ^87^Sr/^86^Sr were possible for each studied individual. The carbon (δ^13^C_coll_) and nitrogen (δ^15^N_coll_) stable isotope analyses were performed on 18 human bone samples recovered from the sites of both studied regions. In addition, to evaluate details of dietary preferences of humans in the CWC communities six animal samples (fragments of long bone diaphysis) were also selected for carbon and nitrogen isotope analyses. However, the investigated graves did not contain local faunal remains, and hence animal samples, broadly contemporary with the studied humans, were taken from other sites located in the Małopolska Upland and closely linked temporally with the CWC. These sites included: Wilczyce (Corded Ware culture), Szarbia (Mierzanowice culture, Early Bronze Age), Smroków (Baden culture, Late Neolithic), and Koszyce (Globular Amphorae culture, Late Neolithic). Unfortunately, no appropriate faunal material from the Subcarpathian region was available for the study.The Sr isotope analyses included human samples obtained from 21 individuals who were excavated from Malżyce and Gabułtów in the Małopolska Upland and from Chłopice, Mirocin, Rozbórz, and Szczytna in the Subcarpathian region. The individuals were selected for sampling based on preserved dental enamel and availability of contextual information. Enamel was taken from first molars (M1) whenever possible and occasionally from premolars (P3, P4). According to time of teeth mineralization they represent signals from childhood and adolescent period. In this study, faunal and environmental samples were also used as a means of establishing local signatures of the investigated areas. Because the investigated CWC features did not contain animal remains, the animal samples came from material excavated in the elaborated areas in younger archaeological sites, e.g. in Gabułtów (Trzciniec culture, older Bronze Age). In other sites of the Małopolska Upland, faunal teeth were taken from sites at Michałowice (Trzciniec culture, older Bronze Age) and Jakuszowice (Przeworsk culture, Roman period) and, in the Subcarpathian region, from a level associated with the Mierzanowice culture (Early Bronze Age) at Mirocin. In overall, tooth enamel samples from various domestic animals were analysed, including 2 samples of cattle, 2 samples of goat/sheep, 4 samples of horse, 2 samples of pig and one sample of dog. We have not collected skeletal remains of the modern fauna because a recent study [38] revealed that modern fauna in Poland is contaminated by anthropogenic Sr, and thus, such samples cannot be unambiguously used to determine the baseline for “bioavailable” ^87^Sr/^86^Sr.

Although a large number of Sr isotope data is available for bedrock and surface waters in south-eastern Poland [39, 40, 41, 42, 43], the Sr isotope composition of rocks exposed or occurring in the shallow subsurface in the investigated areas remains unknown. Therefore, to properly define the isotopic composition of the environmental background, Pleistocene loess deposits and Miocene claystones, which constitute the local bedrock, were collected. Although there is no doubt that the atmospheric waters (rain, snow) during the Final Neolithic period yielded ^87^Sr/^86^Sr values around 0.7092 (see [44]), a single sample of rainwater was measured to check whether the atmospheric waters in southwestern Poland contain any significant amounts of dust derived from natural sources.

## Methods

Collagen extracted for AMS ^14^C dating in the Poznań Radiocarbon Laboratory was transferred to the Isotope Dating and Environment Research Laboratory at the Institute of Geological Sciences of the Polish Academy of Sciences in Warsaw for carbon (δ^13^C_coll_) and nitrogen (δ^15^N_coll_) stable isotope analyses. Stable isotopic composition was determined using a Thermo Flash EA 1112HT elemental analyzer connected to a Thermo Delta V Advantage isotope ratio mass spectrometer in a Continuous Flow system. Isotope ratios were reported as delta (δ) values and expressed relative to VPDB for δ^13^C_coll_ and to atmospheric nitrogen for δ^15^N_coll_. Delta values were normalized to a calibration curve based on international standards USGS 40, USGS 41, IAEA 600. Two bone samples were sent to the ^14^CHRONO Centre for Climate, Environment, and Chronology at Queen's University in Belfast for radiocarbon dating and stable isotope measurements. The Sr isotope analyses were carried out in the Isotope Laboratories at Adam Mickiewicz University (Poznań, Poland) and the University of Copenhagen (Denmark). In Poznań, the sample material comprised human and animal tooth enamel, sediments and rainwater. The procedure included chemical separation of Sr and measurements of Sr isotope ratios. The mechanically isolated enamel was cleaned in an ultrasonic bath in ultrapure water to remove the sediment particles. Afterwards, about 10 mg of powdered enamel was treated sequentially with 0.1 ultrapure acetic acid (5 times) to eliminate the diagenetic Sr contamination, according to the procedure described by Dufour et al. [45]. Subsequently, the samples were dissolved on a hot plate (~100°C overnight) in closed PFA vials using 1 N HNO_3_. The powdered sediment samples (~60 mg) were dissolved on a hot plate (~100°C, three days) in closed PFA vials using a mixture of concentrated hydrofluoric and nitric acid (4:1). About 15 ml of the rainwater sample was evaporated on a hot plate in PFA vials. Subsequently, 340 ml of 2N HNO_3_ was added to the sample and left overnight to equilibrate. The miniaturized chromatographic technique developed by Pin et. al. [46] and modified by Dopieralska [47] was applied for Sr separation. The strontium was loaded with a TaCl_5_ activator on a single Re filament and was analysed in dynamic multi-collection mode on a Finnigan MAT 261 mass spectrometer. Over the course of this study, the NBS 987 Sr standard yielded ^87^Sr/^86^Sr = 0.710229 ± 10 (2σ, n = 34). The measured ratios were normalized to a nominal value of 0.710240 for the standard NBS 987. The total procedure blanks were less than 80 pg.

In Copenhagen, the enamel’s surface was cleaned mechanically, after which the tooth enamel was sampled with a diamond bit. A few milligrams of enamel powder were dissolved in 7 ml Teflon beakers in a 1:1 solution of 0.5 ml 6 N HCl and 0.5 ml 30% H_2_O_2_. The samples dissolved within a few minutes, after which the solutions were dried on a hotplate at 80°C. Subsequently, the enamel samples were placed in a few drops of 3N HNO_3_ and then loaded onto disposable 100 μl pipette tip extraction columns, into which we fitted a frit that retained a 0.2 ml stem volume of intensively pre-cleaned mesh 50–100 SrSpec chromatographic resin. The elution recipe essentially followed that by Horwitz et al. [48]. Samples were loaded with a Ta_2_O_5_-H_3_PO_4_-HF activator on a single Re filament and measured in a dynamic collection mode on a VG Sector 54 IT mass spectrometer. Over the course of this study, the NBS 987 Sr standard was typified by a ^87^Sr/^86^Sr ratio of 0.710241 ± 11 (2σ, n = 5).

## Geological setting

**Fig 4:**
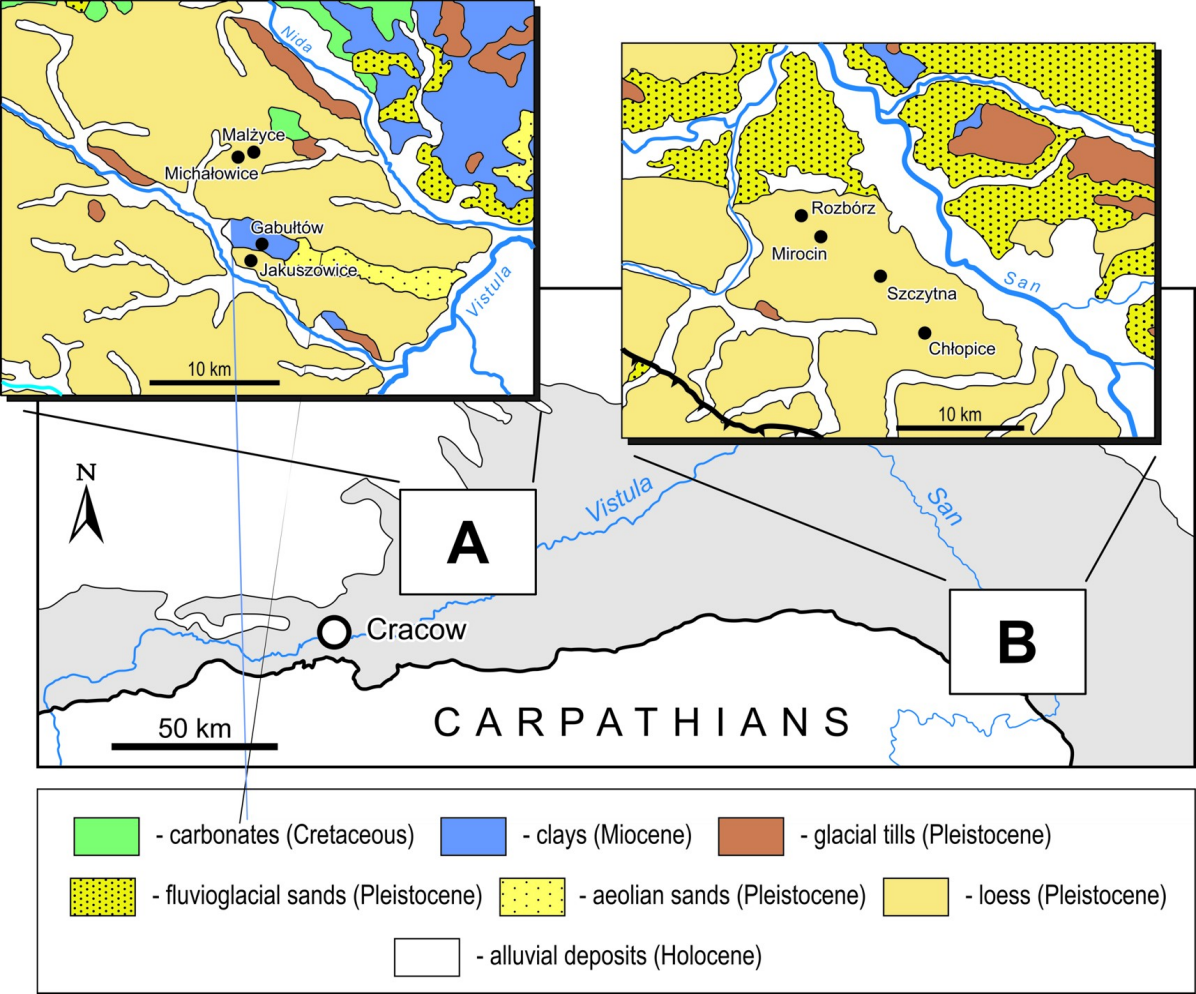
Schematic map of south-eastern Poland with the location of settlement areas of the Corded Ware culture in the Małopolska Upland (A) and the Subcarpathian region (B) investigated during the present study. The main geological units, the Carpathians and the Carpathian Foredeep (grey shading), are indicated. Detailed geological maps with the location of the investigated sites include information from the Geological Map of Poland [51].

Although both areas under study in the Małopolska Upland and Subcarpathian region are situated approximately 130 km from each other, they share a nearly identical geological background. Both are located within a foredeep basin that developed due to thrust loading of the Central European lithospheric plate by the Carpathian orogen during the Miocene times [49, 50]. The Carpathian Foredeep (Fig 4) is predominantly filled with monotonous and very thick argillaceous sediments.

The topmost part of the Miocene sequence usually consists of claystones (Krakowiec Beds). The investigated CWC sites are in areas where these rocks are overlain by a Pleistocene sequence composed of loess and locally of glacial tills and fluvioglacial sands. The loess cover, which forms a typical plateau landscape, is generally only a few metres in thickness. However, locally, it has been removed by erosion, and the Miocene claystones are exposed (Fig 4), for example at the Gabułtów site. The loess plateau is cut by rivers and streams. Their valleys are filled with Holocene alluvial sediments, mostly clays with intercalations of sands, muds and peats.

## Radiocarbon dating

**Fig 5:**
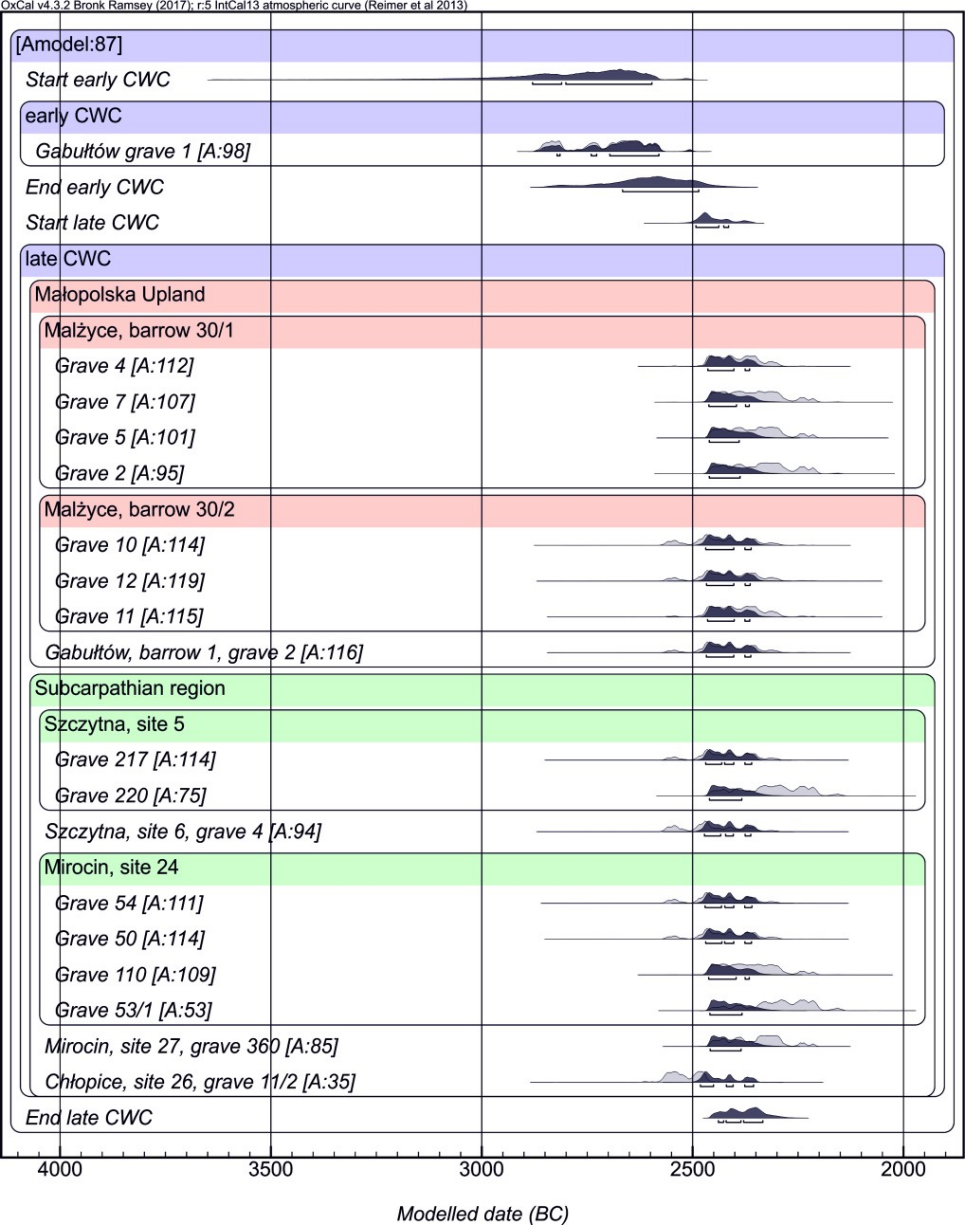
Diagram calibration of radiocarbon dating measurements.

Radiocarbon dating of bone material revealed that the analysed individuals in both regions lived almost simultaneously and dated graves to the younger stage of the CWC (Table 1 and Fig 5).

**Table 1:** Results of radiocarbon dating and isotope analyses of human tissues.

Site/feature, individual	Lab no.	^14^C (BP)	^cal BC 1δ^	δ^13^C_coll_	δ^15^N_coll_	C%	N%	C/N	tooth	87Sr/86Sr	age (years)	sex
Małopolska Upland												
Malżyce 30, t. 1/2	Poz-59407	3860 ± 35	2454–2418 (15.4%) 2407–2376 (14.6%) 2350–2286 (35.9%) 2246–2244 (1.1%) 2238–2236 (1.1%)	–19.4	11.0						30–40	M
Malżyce 30, t. 1/4	Poz-90765	3910 ± 30	2466–2401 (44.1%) 2382–2348 (24.1%)	–19.6	10.1	50.1	18.1	3.22			50–55	F
Malżyce 30, t.1/5	Poz-90767	3870 ± 30	2454–2418 (18.0%) 2407–2376 (16.9%) 2350–2293 (33.3%)	–19.3	10.1	40.8	14.9	3.19			6–7	?
Malżyce 30, t. 1/7	Poz-90768	3875 ± 35	2454–2418 (17.6%) 2408–2374 (16.5%) 2368–2297 (34.1%)	–19.8	10.2	47.5	17.6	3.16	UP3	0.710282 ± 10	20–50	?
Malżyce 30, t. 2/ 10	Poz-27990	3940 ± 40	2548–2540 (3.0%) 2489–2400 (47.7%) 2382–2348 (17.5%)	–19.4	10.6				LP4	0.709538 ± 17	40–45	M
Malżyce 30, t. 2/ 11	Poz-27991	3910 ± 35	2468–2391 (43.8%) 2386–2346 (24.4%)	–20.2	12.2	43.8	15.9	3.22			1–1.5	?
Malżyce 30, t. 2/ 12	Poz-27992	3930 ± 40	2477–2393 (46.6%) 2386–2346 (21.6%)	–20.2	9.0				LP3	0.709664 ± 16	11–12	?
Gabułtów 1/1	Poz-9451	4115 ± 30	2855–2812 (20.7%) 2746–2725 (8.9%) 2697–2620 (38.6%)	chalcoral					UP3	0.710392 ± 15	20–30	M
Gabułtów 1/2, 1	Poz-9452	3930 ± 35	2476–2398 (46.9%) 2383–2347 (21.3%)	–19.8	10.2	39.1	14.3	3.20	LM1	0.710272 ± 10	40–50	M
Gabułtów 1/2, 2									UM1	0.709737 ± 10	6–7	?
Gabułtów 1/2, 3									UM1	0.709561 ± 8	4–5	?
Subcarpathian region												
Szczytna 5/217	Poz-90878	3935 ± 35	2481–2400 (48.4%) 2382–2348 (19.8%)	–20.1	10.5	46.9	16.8	3.27	UM1	0.710598 ± 9	16–18	F?
Szczytna 5/220	UB-28881	3845 ± 37	2430–2424 (2.2%) 2402–2381 (7.8%) 2348–2274 (37.1%) 2256–2208 (21.1%)	–20.2	11.2			3.19	LP4	0.710933 ± 14	40–50	M
Szczytna 6/4	UB-28880	3951 ± 37	2564–2533 (16.3%) 2494–2450 (32.9%) 2420–2404 (6.9%) 2378–2350 (12.2%)	–20.0	11.0			3.20	P	0.710872 ± 15	25–30	M
Szczytna 6/56									UM1	0.710374 ± 12	25–30	M
Szczytna 6/84									LM1	0.710884 ± 10	5–6	?
Szczytna 6/150									UM2	0.711271 ± 9	14–16	M
Mirocin 24/54	Poz-54038	3940 ± 35	2546–2541 (2.0%) 2488–2431 (36.3%) 2424–2401 (12.3%) 2381–2348 (17.7%)	–20.2	10.7	42.6	15.1	3.29	LM1	0.709367 ± 16	50–60	M
Mirocin 24/110, 1	Poz-90885	3880 ± 35	2456–2416 (20.7%) 2410–2334 (38.7%) 2324–2306 (8.8%)	–20.0	10.9	55.3	20.5	3.16	LM1	0.710454 ± 9	40–50	M
Mirocin 24/50	Poz-90880	3935 ± 35	2481–2400 (48.4%) 2382–2348 (19.8%)	–20.0	11.4	45.6	16.1	3.31	UM1	0.710537 ± 12	40–50	M
Mirocin, 24/53, 1	Poz-91015	3835 ± 35	2344–2206 (68.2%)	–19.3	13.5	53.5	19.3	3.24			3–4	?
Mirocin 27/360, 1	Poz-54043	3870 ± 35	2454–2418 (17.2%) 2408–2374 (16.4%) 2368–2362 (2.6%) 2354–2292 (32.0%)	–20.4	9.5	44.4	15.8	3.28	LM1	0.708881 ± 14	50–60	M
Mirocin 27/360, 2	Poz-90887	3860 ± 30	2454–2418 (14.8%) 2406–2376 (14.8%) 2350–2286 (38.6%)	–20.3	10.6	49.7	18.3	3.17	LM1	0.709021 ± 15	40–50	F?
Rozbórz, 42/2006									UM1	0.709743 ± 10	18–22	M
Chłopice 26/11, 1									LM1	0.710495 ± 10	11–12	?
Chłopice 26/11, 2	Poz-90881	3985 ± 35	2566–2524 (41%) 2497–2471 (27.2)	–20.0	11.0	19.0	51.6	3.17	LP3	0.710853 ± 10	14–15	?

t. = tumulus

Sex: M = Male; F = Female; ? = Unknown.

Teeth types: LM1 = lower first molar; UM1 = upper first molar; UM2 = upper second molar; P = premolar; LP3 = lower third premolar; LP4 = lower fourth premolar; UP3 = upper third premolar.

However, single charcoal dating for grave 1 at Gabułtów (Poz-9451: 4115±30 BP) assigned its age to the older mound period which corresponds to 2855–2620 BC interval and it is compatible with the other dates from barrow graves from south-eastern Poland. The sample was taken from the burnt wooden construction of the grave. The measurements were carried out using the accelerator technique (AMS) at the Poznań Radiocarbon Laboratory (17 samples) and at the Belfast laboratory (2 samples). The δ^15^N _coll_ values of the dated samples in the range of 9.5–13.5 do not indicate older ^14^C ages due to the freshwater effect. The calendar age was set using the OxCal v4.3.2 calibration programme of Ch. Bronk Ramsey from 2017 [52]. The vast majority of results for the younger phase, including all from the Małopolska Upland, indicate the 2500–2300 BC range. The weight lies in the time around 2400 BC, to which the age of the most of samples can be assigned with the highest probability. A slightly older age—2566–2471 BC, was obtained only for a single grave at Chłopice. In contrast, a slightly younger age refers to grave no. 53 from Mirocin 24, where the combined date from these features indicates with the greatest probability on the range 2300–2200 BC. The measurement results are generally consistent with the series of datings obtained for similar CWC graves from other regions of Małopolska [53, 54, 55].

## Results

**Fig 6:**
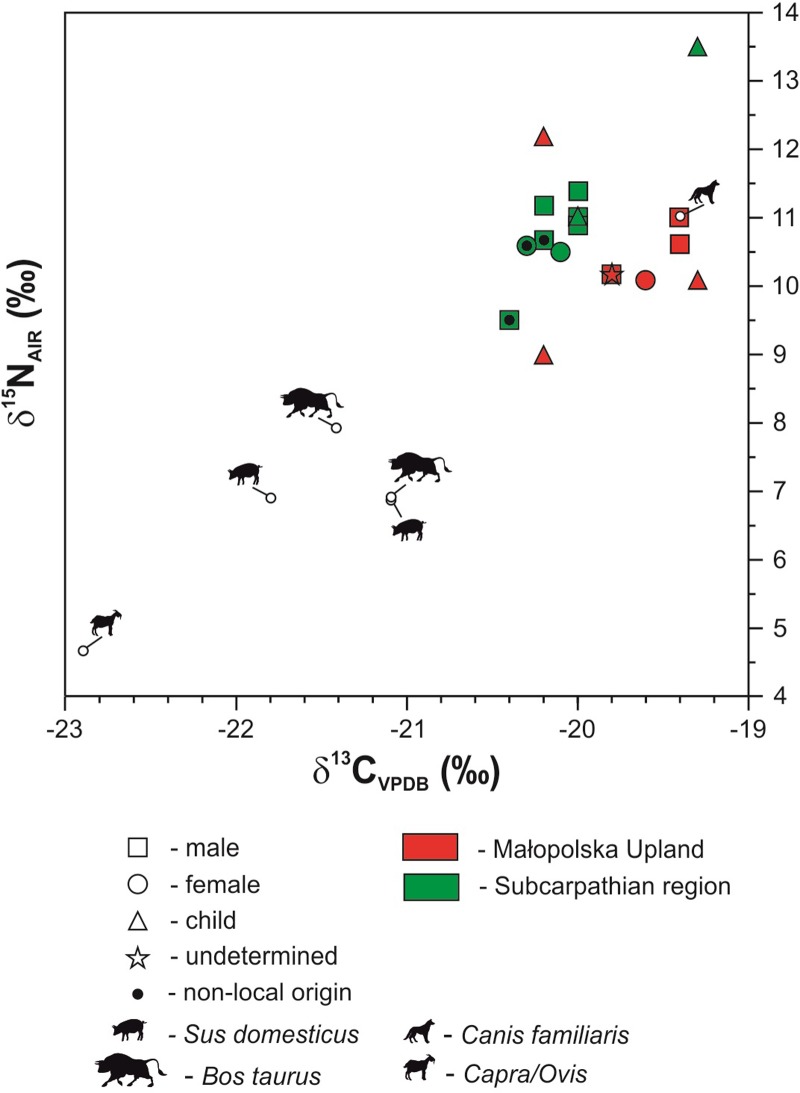
Plot of δ^13^C _coll_ and δ^15^N _coll_ values for humans and animals.

In all 19 bone samples, the collagen was well preserved; its C/N ratios, below 3.6, show no significant alteration of in vivo isotopic signatures [56]. The results of the carbon and nitrogen isotope analyses are presented in Fig 6 and are listed in Tables 1 and 2.

**Table 2:** Results of radiocarbon dating and stable isotope analyses of animal bones.

Site/feature, individual	Lab no.	^14^C (BP)	^cal BC 1δ^	δ^13^C_coll_	δ^15^N_coll_	C%	N%	C/N	age	species
Smroków 17/8	Poz-34690	4010 ± 40	2572–2512 (48.2%) 2504–2478 (20.0%)	-22,9	4,7	44,6	16	3,24	adult	*Capra/Ovis*
Smroków 17/7	Poz-34688	4360 ± 40	3015–2916 (68.2%)	-21,1	6,9	41,9	15,2	3,23	adult	*Bos taurus*
Smroków 17/14	Poz-34691	4570 ± 40	3487–3472 (5.5%) 3372–3330 (30.9%) 3216–3182 (16.6%) 3158–3124 (15.2%)	-21,1	6,9	42,5	15,5	3,2	adult	*Sus domesticus*
Szarbia 9/1, V	Poz-34685	3810 ± 35	2298–2198 (63.6%) 2164–2152 (4.6%)	-21,4	7,9	43	15,8	3,18	adult	*Bos taurus*
Wilczyce 15	Poz-59139	4030 ± 35	2579–2488 (68.2%)	-19,4	11	-	-	-	adult	*Canis familiaris*
Koszyce 3/506, 1	Poz-47437	4125 ± 35	2858–2809 (21.1%) 2752–2722 (13.1%) 2702–2624 (34.1%)	-21,8	6,9	-	-	-	adult	*Sus domesticus*

**Fig 7:**
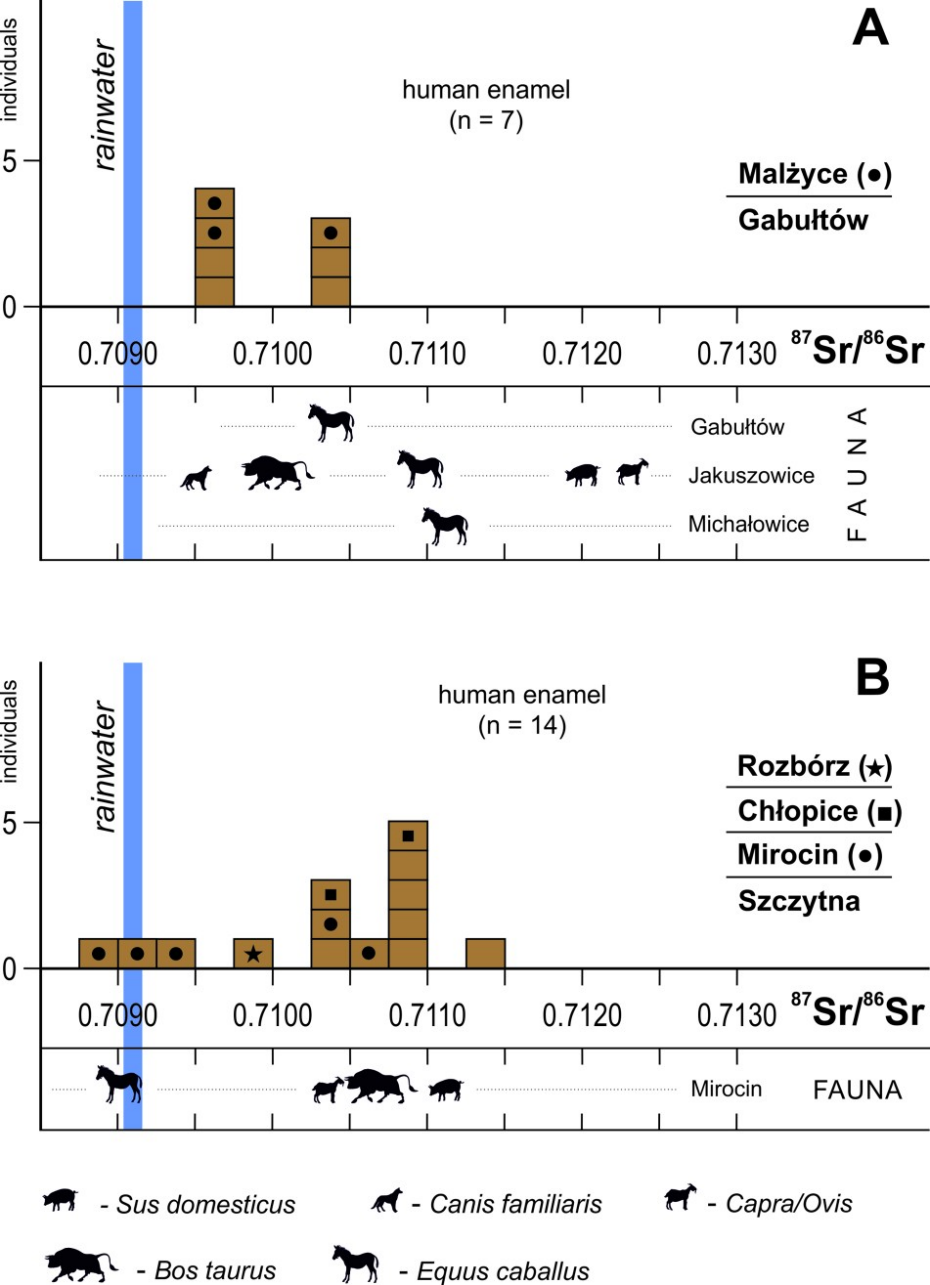
Strontium isotope ratios in Final Neolithic human and animal enamel in the Małopolska Upland (A) and in the Subcarpathian region (B). Each faunal silhouette indicates a single sample. The Sr isotope composition of rainwater, the most non-radiogenic element of the local environment, is indicated.

The human δ^13^C_coll_ values in the Subcarpathian region vary within a narrow range, between –20.4 and –19.3‰. In contrast, their nitrogen isotope compositions (δ^15^N_coll_) exhibit larger variations, from 9.5 to 13.5‰; this wide range, however, is due to the occurrence of two individuals at Mirocin that yield the highest and lowest signatures, respectively. Without these outliers, the remaining group is characterized by a narrow range of δ^15^N_coll_ values, between 10.2 and 11.4‰. In the Małopolska Upland, the δ^13^C_coll_ values of individuals are within an almost identical range (–20.2 to –19.3‰) as in the Subcarpathian region. Their nitrogen isotope values (δ^15^N_coll_) are also similar, ranging from 9.0 to 12.2‰. Here, the lowest and highest values, which expand the range, are from two infants, while the values of all adults range between 10.1 and 11.0‰. Overall, in both studied regions, there are no significant differences in collagen stable isotope values that are dependent on the age at death (Table 1). However, it is important to note that the most divergent isotopic signatures, which are remarkably different from those of the rest of the population, belong to individuals who died in childhood (Fig 6). When compared to humans samples, all domestic animals from the Małopolska Upland, with the exception of a dog from Wilczyce, demonstrate depleted levels of both δ^13^C_coll_ and δ^15^N_coll_ values. The isotopic offsets between the animal and human collagen samples are about 2‰ and 3−4‰ for carbon and nitrogen, respectively. The Sr isotope data from the human and animal enamel are presented in Tables 1 and 3 and Fig 7.

**Table 3:** Strontium isotope ratios for animal enamel samples from the investigated sites of south-eastern Poland.

Locality, site	Feature	Species	^87^Sr/^86^Sr
Mirocin, 27	292	*Bos taurus*	0.710694 ± 10
	367	*Capra/Ovis*	0.710314 ± 10
	367	*Equus caballus*	0.709077 ± 10
	245	*Sus domesticus*	0.711123 ± 12
Michałowice, 1	57	*Equus caballus*	0.711168 ± 10
Gabułtów, 1	4	*Equus caballus*	0.710256 ± 10
Jakuszowice, 2	1369	*Bos taurus*	0.709958 ± 10
	1760	*Capra/Ovis*	0.712328 ± 9
	1760	*Equus caballus*	0.711084 ± 10
	1430	*Canis familiaris*	0.709552 ± 11
	1258	*Sus domesticus*	0.712117 ± 15

In the Małopolska Upland, the ^87^Sr/^86^Sr signatures of humans are grouped in two clusters, one around 0.7096 and another around 0.7104. This pattern contrasts with the wide range of ^87^Sr/^86^Sr values of the animal teeth measured within this study, which range from 0.7095 to 0.7123. However, this range is defined by animal material from the Roman period collected at Jakuszowice, whereas the single horse found at Gabułtów coincides perfectly with the isotopic composition of some humans in this locality. In the Subcarpathian region, human tooth enamel ^87^Sr/^86^Sr values vary within a wide range, from 0.7089 to 0.7114, with a mode that is not normally distributed. There is a main cluster comprising more radiogenic signatures, from 0.7104 to 0.7114, which includes all individuals from Szczytna and Chłopice and two Mirocin males. Another cluster consists of three individuals with less radiogenic ^87^Sr/^86^Sr values (all from Mirocin) and a single male from Rozbórz with an in-between Sr signature of 0.7097. Although our few animal enamel data from the Subcarpathian region cannot be considered statistically representative, most of them (except signature for one horse from Mirocin) correspond well with the distribution mode of obtained for humans (Fig 7).

**Fig 8:**
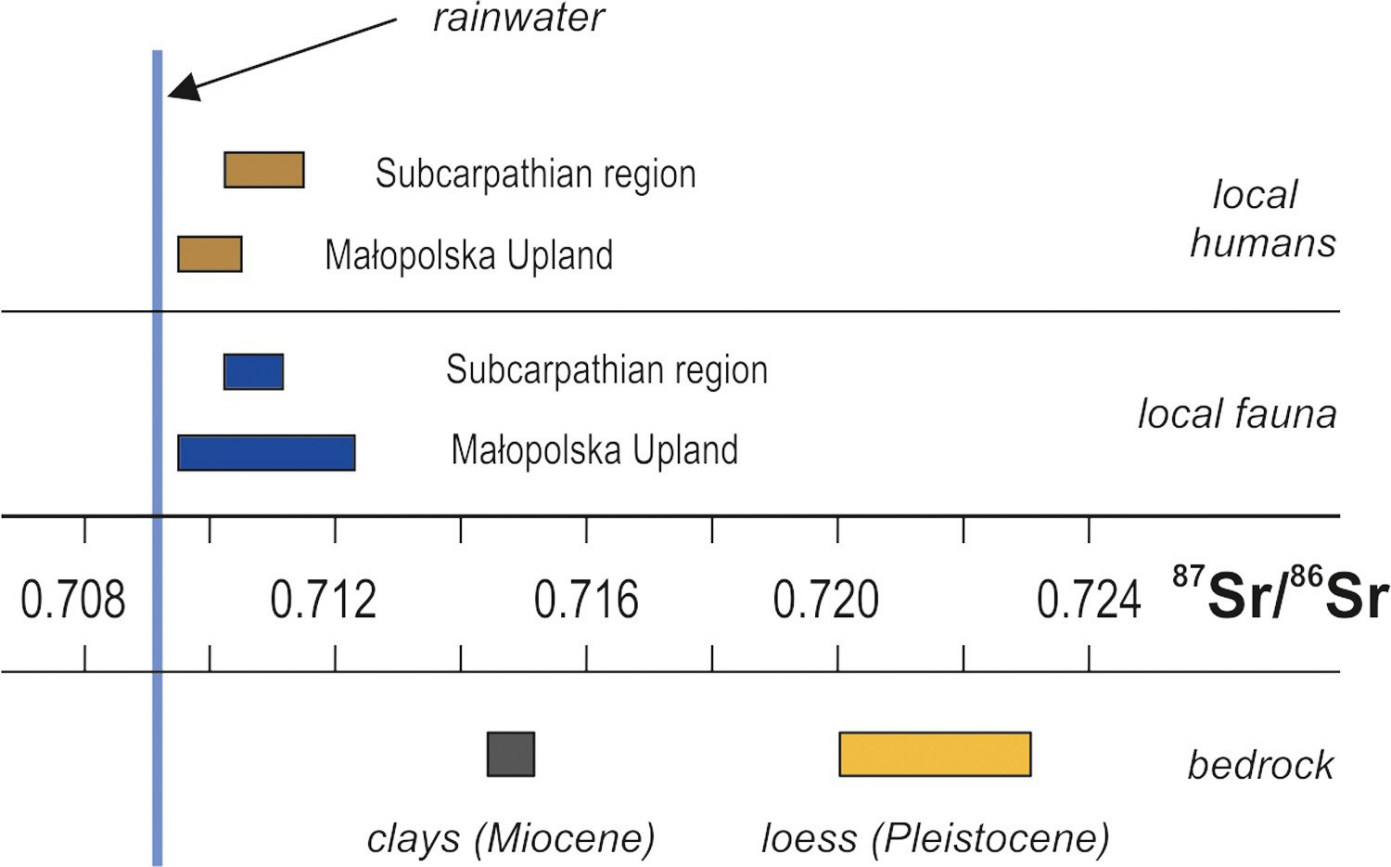
Ranges of ^87^Sr/^86^Sr ratios in Final Neolithic human and animal enamel from the Małopolska Upland and the Subcarpathian region, along with values for local geological bedrock and rainwater in the studied areas.

The results of the Sr isotope analyses show that the surface bedrock in both study areas exhibits a similar Sr isotope composition with relatively high radiogenic ^87^Sr/^86^Sr ratios (Table 4 and Fig 8).

**Table 4:** Strontium isotope signatures (^87^Sr/^86^Sr) of bedrock and rainwater in the studied areas of south-western Poland.

Sample	Locality	Location		^87^Sr/^86^Sr
		Latitude	Longitude	
Loess (Pleistocene)	Odonów	50°14ʹ34.11ʺ N	20°29ʹ19.39ʺ E	0.722955 ± 10
Loess (Pleistocene)	Kolosy	50°19ʹ09.39ʺ N	20°36ʹ56.50ʺ E	0.723484 ± 21
Loess (Pleistocene)	Radymno	49°57ʹ19.34ʺ N	22°48ʹ29.98ʺ E	0.720016 ± 12
Claystone (Miocene)	Hadykówka	50°19ʹ38.76ʺ N	21°44ʹ27.15ʺ E	0.715182 ± 10
Claystone (Miocene)	Odonów	50°14ʹ34.11ʺ N	20°29ʹ19.39ʺ E	0.714809 ± 10
Claystone (Miocene)	Kolosy	50°19ʹ39.65ʺ N	20°37ʹ53.38ʺ E	0.714447 ± 10
Rainwater	Odonów	50°14ʹ34.11ʺ N	20°29ʹ19.39ʺ E	0.709290 ± 10

Pleistocene loess deposits, with signatures from 0.7200 to 0.7234, are more radiogenic than the underlying Miocene claystones, which have uniform ^87^Sr/^86^Sr ratios around 0.7150. Compared to loess deposits in the Alpine Foreland and southeastern Europe [57, 58], the investigated loess in southeastern Poland is characterized by a more radiogenic composition. Our data confirm an earlier report by Svensson et al. [59] who presented a strong radiogenic ^87^Sr/^86^Sr value (0.734) for loess deposited in western Ukraine. As expected, rainwater collected in the Małopolska Upland has a Sr isotope signature close to 0.7092, which is the composition of modern ocean water [60]. Hence, there is no indication for any noticeable influence of continental dust sources.

In the Subcarpathian region, the investigated sites are located not far away from valleys filled up with Holocene alluvial sediments, composed of detrital material eroded from the Carpathian Mountains. The Sr isotope composition of these sediments has not been studied so far. The Carpathians are predominantly built of monotonous sequences of various Cretaceous and Paleogene clastic rocks. Their siliciclastic composition [61] points to radiogenic Sr isotope signatures higher than 0.71. Hence, it is likely that the Holocene alluvial sediments in the drainage valleys of the eastern Carpathians have Sr isotope signatures similar to those of the investigated Miocene claystones and Pleistocene loess deposits.

## Discussion

The 3rd millennium BC was a time of significant cultural and social changes, which led to the emergence of Bronze Age societies. Understanding these change processes requires interdisciplinary investigations that combine standard archaeological and anthropological studies with modern analytical techniques. Among the latter are methods that utilize stable and radiogenic isotopes in soft or mineralized tissues of humans and animals to provide constraints on dietary preferences, provenance and mobility (for review see [9, 62, 63, 64, 65, 66]); thus, this information cannot be directly gathered alone from the archaeological context of the studied materials.

The carbon and nitrogen isotope composition of bone collagen enables the reconstruction of diet and nutritional customs of prehistoric humans; therefore, the method is routinely applied in archaeological research [1, 65, 66, 67]. Experimental studies have shown that the δ^13^C values of collagen are at inland sites generally linked to those of the protein fraction of the diet [68]. The δ^15^N_coll_ values of humans are higher than those of their average diet. However, they cannot be considered as absolute proxies for the diet since the δ^15^N _coll_ values of plants can be influenced by environmental conditions [69].

**Fig 9:**
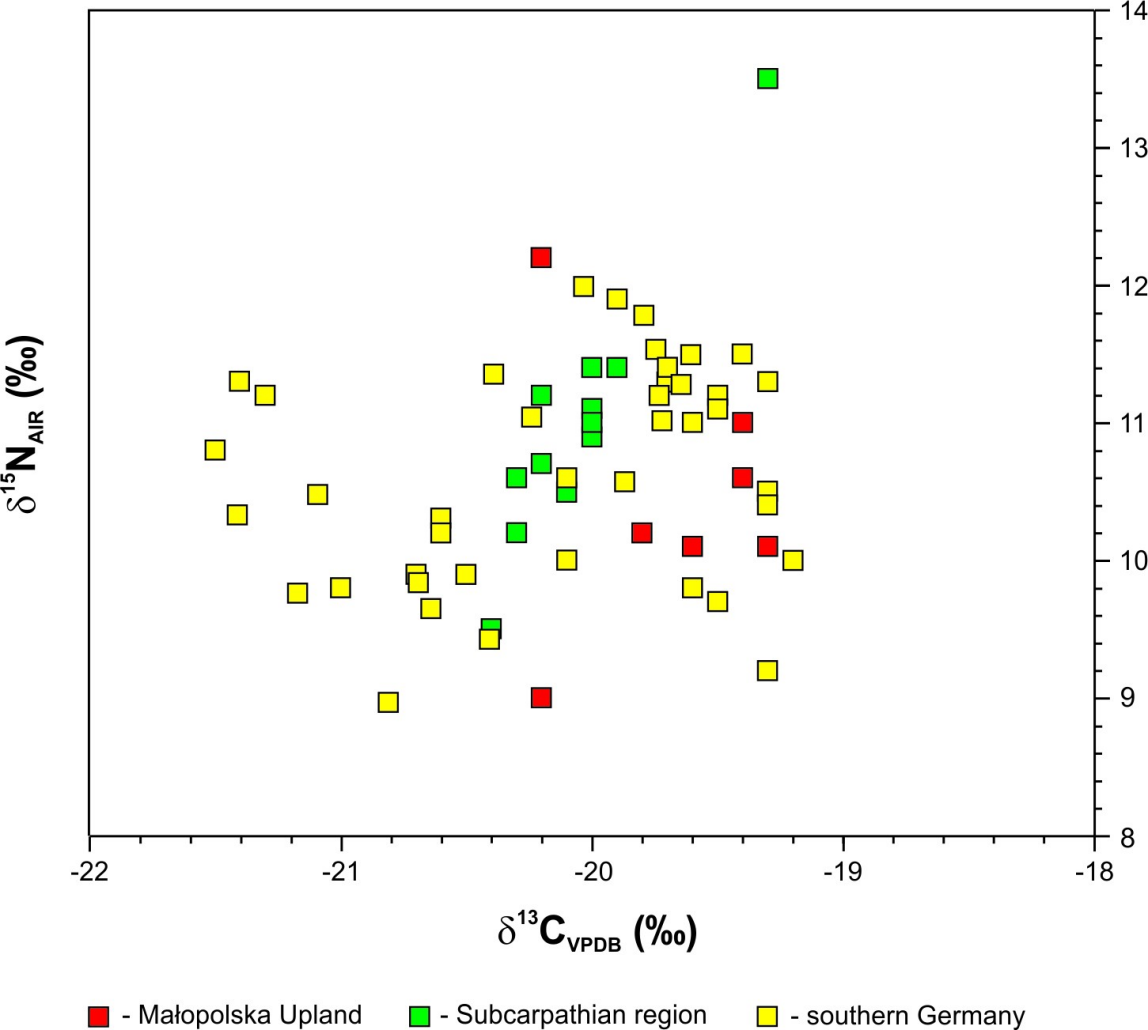
Plot of δ^13^C versus δ^15^N values for CWC humans from southwestern Poland compared to southern Germany populations of this culture [73].

The narrow and similar ranges of human δ^13^C_coll_ values around –20‰ recognized in the Małopolska Upland and in the Subcarpathian region are typical for humans living in inland temperate environments that have a terrestrial diet based on C_3_-plant and animal resources (for comparison, see [70, 71]. At the same time, the results from both regions are within the range of variability set by the relatively large amount of data from CWC individuals from southern Germany (Fig 9) [72, 73].

These values compared to animal data (Fig 6) indicate that herbivore meat and/or dairy protein were present in the diet of the studied CWC human individuals but plant food sources appear to have constituted the main portion of it. It is remarkable, however, that the adults in the Małopolska Upland have systematically higher δ^13^C_coll_ values by about 0.5‰ compared to adults in the Carpathian region (Table 1 and Fig 6). The difference suggests a higher proportion of animal protein in the diet, but can also be attributed to regional differences in land-cover (wooded vs. unwooded areas) or in climatic conditions [74]. The δ^15^N_coll_ values, ranging from about 10‰ to 11.4‰ indicate that freshwater fish did not contribute significantly to the diet in both study areas. The offsets of about 2‰ for carbon and 3−4‰ for nitrogen, between humans and animals (herbivores and the omnivorous pigs), represent characteristic trophic enrichment values for one trophic level shift [e.g., 8, 75]. The prehistoric dog most probably consumed food similar to that of humans is common to several Mesolithic and Neolithic communities [e.g., 76, 77]. The fact that two children (Mirocin site 24, feature 53, individual 1 and Malżycesite 30, tumulus 2, feature 11) yield the highest δ^15^N_coll_ values in the studied population (Fig 6) appears to record a ‘weaning signal’ from consumption of milk and indicates presumably that weaning phase lasted until their death. The isotopic composition of bone and dentine (dm1- deciduous first molar, dm2—deciduous second molar and M1 –permanent first molar) collagen recovered from children skeletons from Malżyce (tumulus 2 individuals 11 and 12) was already investigated during an earlier study, which focused on breastfeeding and weaning practices in Małopolska during the Middle Neolithic to Early Bronze period [78]. A complex analysis conducted for a child (no. 12) who died at approximately 11–12 years of age showed that δ^15^N_coll_ drops by 1.5‰ between the dm2 and M1 dentine samples in addition to a further 0.7‰ lower for the bone sample. These results suggest slow and gradual weaning that was not completed until after the M1 dentine started forming early in the child’s fourth year. For the bone collagen of this individual, we received even lower δ^15^N value (9‰). It seems that after weaning, this child began to consume less animal protein than the adults. However, because this individual died at a young age, his low δ^15^N_coll_ level could be connected to a physiological condition. Katzenberg and Lovell [79] argued that the δ^15^N ratio in collagen is affected by a variety of factors, including nutritional stress or ill health.

The low δ^15^N_coll_ signature of 9.5‰, discovered in the male individual buried in grave no. 360 (site 27) at Mirocin, is the lowest nitrogen value among all adults in the investigated areas. This person belongs to individuals identified in the Subcarpathian region as non-locally born (see discussion below). The reason behind this difference could simply reflect his preferred diet and potential higher consumption of plant food sources. Most probably his eating habits were different from that of other individuals. It remains unclear to what extent other factors, such as environmental stress or traumatic events, may have contributed to this distinction. Previous research showed that various pathological processes, such as cirrhosis, may reduce the level of nitrogen [80]. The male skeleton in grave no. 360 did not display any other pathological lesions beyond fully healed cranial trauma, yet with the time of injury remaining unknown it is not possible to establish a link between the cranial lesion and the lowered δ^15^N_coll_ value.

Isotopic measurements of the environmental samples revealed that the overall Sr isotope systems in both studied areas are almost identical. Both display a distinct bipolar pattern with the bedrock, which comprises the most radiogenic components, and rainwater, which constitutes the less radiogenic element of the environment. This implies a Sr isotope composition of surface waters (streams), with signatures between rainwater and bedrock. The Sr^87^/^86^Sr values of humans and animals in the Małopolska Upland (Fig 7) are consistent with the local environmental background. More radiogenic signatures of some animals (pig, caprine), with signatures closer to those of the bedrock, are probably due to herding on the loess plateau, in contrast to cattle and horses, which presumably grazed in the valleys of streams. However, it cannot be ruled out that animals from the Przeworsk culture settlement at Jakuszowice could have been imported. The local origin of all studied individuals in the Małopolska Upland correlate well with the associated records of archaeological materials in the sites, which show only local pottery vessels and lithic industry [22].

Although most human ^87^Sr/^86^Sr values in the Subcarpathian region lie within a mixing space between rainwater and local bedrock, the local origin of all investigated individuals seems to be unlikely. It seems that the main cluster of ^87^Sr/^86^Sr values of humans, from 0.7104 to 0.7114, represents local Sr signatures and thus all individuals from Szczytna and Chłopice, and two Mirocin males can be recognized as “locals”. Their local origin is compatible with the indigenous ceramic identified in grave inventories [81]. In contrast, two males and a female at Mirocin (from grave 54, site 24 and double grave 360, site 27), having unradiogenic Sr signatures between 0.7089 and 0.7094, are doubtlessly “non-locals” who did not reside in the Subcarpathian region during childhood. Their Sr signatures are close to the rainwater composition, but rainwater, with its extremely low Sr concentration, generally below 1 ppb [38, 82] and cannot constitute the main Sr source for humans and animals. These signatures attest to the presence of an unradiogenic bedrock component in the local environment, with ^87^Sr/^86^Sr values lower than that of rainwater. In the Subcarpathian region, around the studied CWC sites, natural sources of Sr (rocks, sediments) that are less radiogenic than rainwater do not occur, which is also why the horse found at Mirocin could not be from the local domestic fauna. Its unradiogenic Sr signature points to origin from an area where marine Neogene (Miocene) carbonate rocks constitute the local bedrock. The nearest areas that meet these conditions are located along the northern and eastern margins of the Carpathian Foredeep (Fig 4), at a distance of more than 70 km from Mirocin.

The signatures of three individuals found at Mirocin, identified as non-local, correspond well with the archaeological context. Rich funeral inventories found in the double niche grave no. 360 (site 27) included vessels, lithic artefacts and copper ornaments. One of the beakers was typical of the Middle Dnieper culture which developed in the basins of Dnieper and Pripyat rivers on the territory of the present-day Belarus and Ukraine [81]. Similarly, grave no. 54 (site 24) contained also a beaker typical of the Middle Dnieper culture, which was among other grave goods of local provenance. The non-local beakers are distinguished by their characteristic typological features and also by the petrographic composition and the structure of the ceramic fabric [83]. Therefore, the origin of the non-local individuals at Mirocin from the areas of the Middle Dnieper culture settlement appears to be likely. Additionally, their Sr isotope signatures are in accordance with unradiogenic Cretaceous and Neogene rocks, which are widely exposed in both areas. These rocks are marine carbonate deposits and thus, their Sr composition is well known. Their ^87^Sr/^86^Sr values range from 0.7073 to 0.7078 and from 0.7083 to 0.7089, respectively [60].

The male buried at Rozbórz, with a Sr signature of 0.7097 (Fig 7), cannot be unambiguously defined as local or non-local. However, his grave was equipped with artefacts made of Jurassic flint, which is common in CWC grave inventories in the Małopolska Upland [22]. Therefore, a foreign origin is suggested, possibly from the northern part of Małopolska.

In summary, the study underlines the importance of the Sr isotope method for understanding human mobility in the CWC communities, indicated only indirectly by the presence of non-local flint artefacts and vessels. The compliance of archaeological and isotopic data reveals additional important aspects of social life and funeral customs. The results allow us to claim that foreign individuals must have been successfully integrated into local CWC communities. Their burials were prepared in a planned way and were richly equipped. Their allochthonous origin was also marked by the addition of foreign inventories.

Last, the study shows that the use of fauna for assessment of the local ^87^Sr/^86^Sr range in the Subcarpathian region, as suggested by Price et al. [14] and Bentley et al. [13], would led to the incorrect conclusions. It is consistent with a tendency recently presented by Grimstead et al. [17] that the “archaeological literature employing ^87^Sr/^86^Sr sourcing has shifted away from utilizing archaeological fauna to derive baselines”. For instance, Maurer et al. [84] showed in their comprehensive isotopic study of archaeological, geological and environmental samples in central Germany that animal tooth enamel and snail shells are not appropriate materials to provide a reliable ^87^Sr/^86^Sr baseline for investigating past human migration. Very recently, Lengfelder et al. [85] presented a model for the prediction of a local Sr isotope signatures based on isotopic data from groundwater, soil, wood, and precipitation because a non-local provenance of archaeological animals can never be excluded beforehand.

The Sr isotope composition of human tissues is first of all the effect of mixing of Sr derived from food and drinking water [86]. The Sr isotope compositions of food and drinking water themselves are also the result of complex mixing processes in the geological and biological environment. Hence, we conclude that the interaction between bedrock and water (atmospheric and surface waters) in the local environment constitutes a decisive process which defines a possible range of ^87^Sr/^86^Sr values generated in plant, animal, and human tissues within a given area (see also [87]).

## Conclusions

The study provided the first insights into the dietary preferences in the CWC communities of south-eastern Poland. Our results suggest that individuals in the Małopolska Upland and in the Subcarpathian region subsisted on a similar omnivorous diet composed of C_3_-based terrestrial plant and animal foods, in which plant food dominated. In both regions, there were no significant gender differences in dietary intake. However, there was a local variation in diet composition between the sites. In the Małopolska Upland, the mix of plant and animal foods contained a higher proportion of the latter. Higher δ^15^N_coll_ values of younger infants are interpreted as representing the effects of weaning.

Sr isotope data indicate that the CWC community in the Małopolska Upland consisted exclusively of the local population. In contrast, in the Subcarpathian region, four individuals (28% of the investigated group) were identified as people who died at a location other than place of their childhood. The Sr signatures of non-local individuals correspond well with associated allochthonous grave inventories of eastern or western provenience.

We conclude also that a detailed Sr isotopic survey of the geological background and its hydrologic elements, rather than that of the fauna, seems to provide conclusive constraints for identification of local and non-local individuals in prehistoric communities.
